# FEA model analysis of the effects of the stress distribution of saddle-type implants on the alveolar bone and the structural/physical stability of implants

**DOI:** 10.1186/s40902-016-0054-4

**Published:** 2016-02-20

**Authors:** Yoon Soo Kong, Jun Woo Park, Dong Ju Choi

**Affiliations:** Department of Dentistry, Hallym University College of Medicine, Gangdong Sacred Heart Hospital, 150, Seongan-ro, 05355 Gangdong-gu, Seoul Korea

## Abstract

**Background:**

As dental implants receive masticatory stress, the distribution of stress is very important to peri-implant bone homeostasis and implant survival. In this report, we created a saddle-type implant and analyzed its stability and ability to distribute stress to the surrounding bone.

**Methods:**

The implants were designed as a saddle-type implant (SI) that wrapped around the alveolar bone, and the sizes of the saddles were 2.5, 3.5, 4.5, and 5.5 mm. The X and Y displacement were compared to clarify the effects of the saddle structures. The control group consisted of dental implants without the saddle design (CI). Using finite element modeling (FEM), the stress distribution around the dental implants was analyzed.

**Results:**

With saddle-type implants, saddles longer than 4.5 mm were more effective for stress distribution than CI. Regarding lateral displacement, a SI of 2.5 mm was effective for stress distribution compared to lateral displacement. ASI that was 5.6 mm in length was more effective for stress distribution than a CI that was 10 mm in length.

**Conclusions:**

The saddle-type implant could have a bone-gaining effect. Because it has stress-distributing effects, it might protect the newly formed bone under the implant.

## Background

Severe resorption of the alveolar bone can occur due to various causes, including long-term edentulous states after tooth loss or tooth extraction due to severe periodontitis or severe trauma, and so on [1]. In these cases, functional and esthetic problems occur but are very difficult to manage. In previous reports, wide diameter implants have obtained larger implant surfaces to contact the bone, and as a result, these implants have shown greater initial stability with effective stress distribution; based on these results, prosthodontic stability has increased [2]. However, the conditions of surgery for implants are sometimes impossible to meet due to anatomical limitations if the alveolar bone has resorbed too much.

Recently, guided bone regeneration (GBR) with titanium mesh has become increasingly common, showing good results. However, all of these studies have been limited to only bone regeneration itself, and there have been no studies of the implants remaining on the implanted sites or the stress distribution of these implants during functional loading. Furthermore, there have also been few studies of extra short implants for extremely resorbed alveolar bone [3, 4].

In this study, the saddle-type implant was studied to replace the root-type implant and to resolve the issue of resorbed alveolar bone by supporting bone regeneration, as well as to observe the biomechanical behavior, especially stress distribution. This new type of implant was designed to wrap around the upper part of the alveolar bone like a saddle, and at the center of saddle, the implant served the purpose of fixing the saddle structure onto the alveolar bone and connecting the implant with prosthodontics.

As dental implants receive masticatory stress, the distribution of stress is greatly important to peri-implant bone homeostasis and implant survival. In this report, we created a saddle-type implant (SI) and analyzed its stability and its ability to distribute stress to the surrounding bone, compared to the conventional type of implant (CI), which did not have the saddle design.

## Methods

### Basic design of the finite element analysis model

Samples for the alveolar bone were obtained using a Master 3D CT X-ray tool (Vatech Korea, Seoul, Republic of Korea), with settings of 90.0 kVp and 30 mA. Through this sample, we obtained information about the basic shape of alveolar bone, its structural characteristics, the thickness of the cortical bone, pattern of the cancellous bone, etc. Additionally, the pattern of implant insertion was checked, and this information was used as a reference for the implant finite element analysis (FEA) model.

The FEA model was constructed as the cortical bone, cancellous bone, and implant. The form of rectangles of alveolar bone was based on computed tomography (CT). The saddle structure of the implant and surrounding bone was constructed tightly for greater accuracy of the data. To interpret the data, tetra and prism elements were used for the FEA model; the node number was 13405, and the element number was 64847 using 3D solid structure, and linear static analysis was performed.

**Fig. 1:**
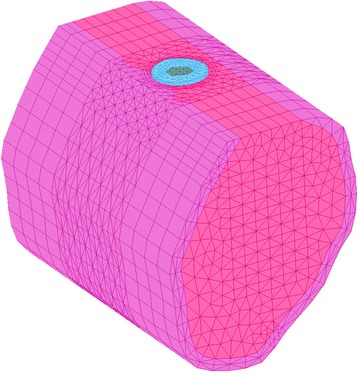
The basic design for the analysis of FEA models

**Fig. 2:**
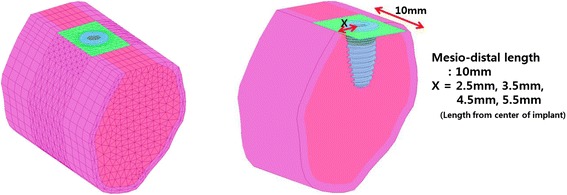
Class I modifications

**Fig. 3:**
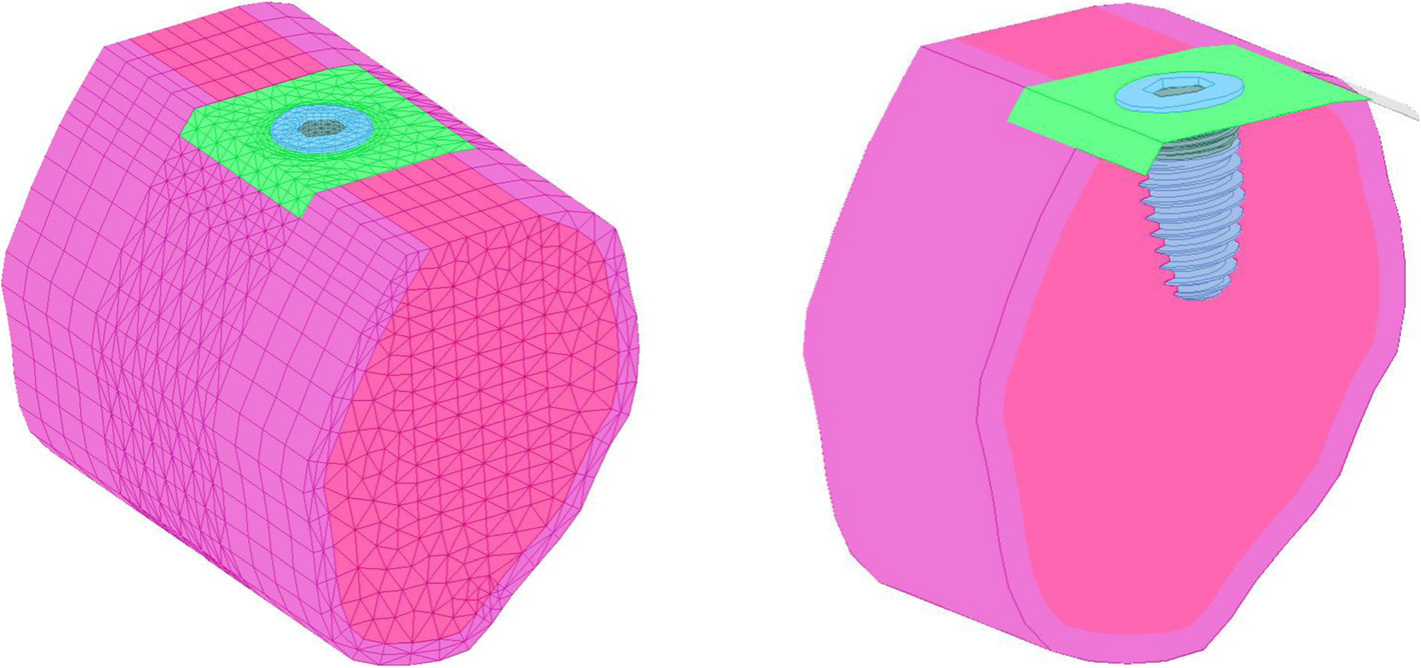
Class II modifications

**Fig. 4:**
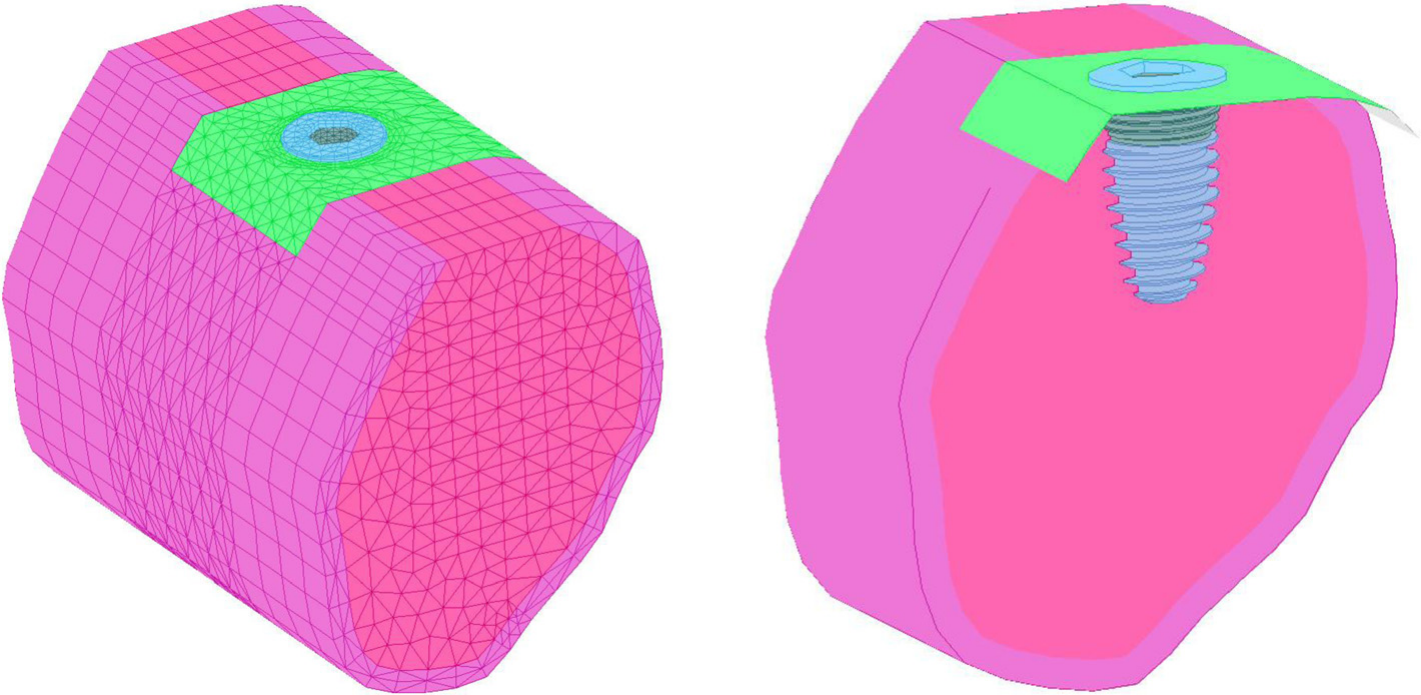
Class III modifications

**Fig. 5:**
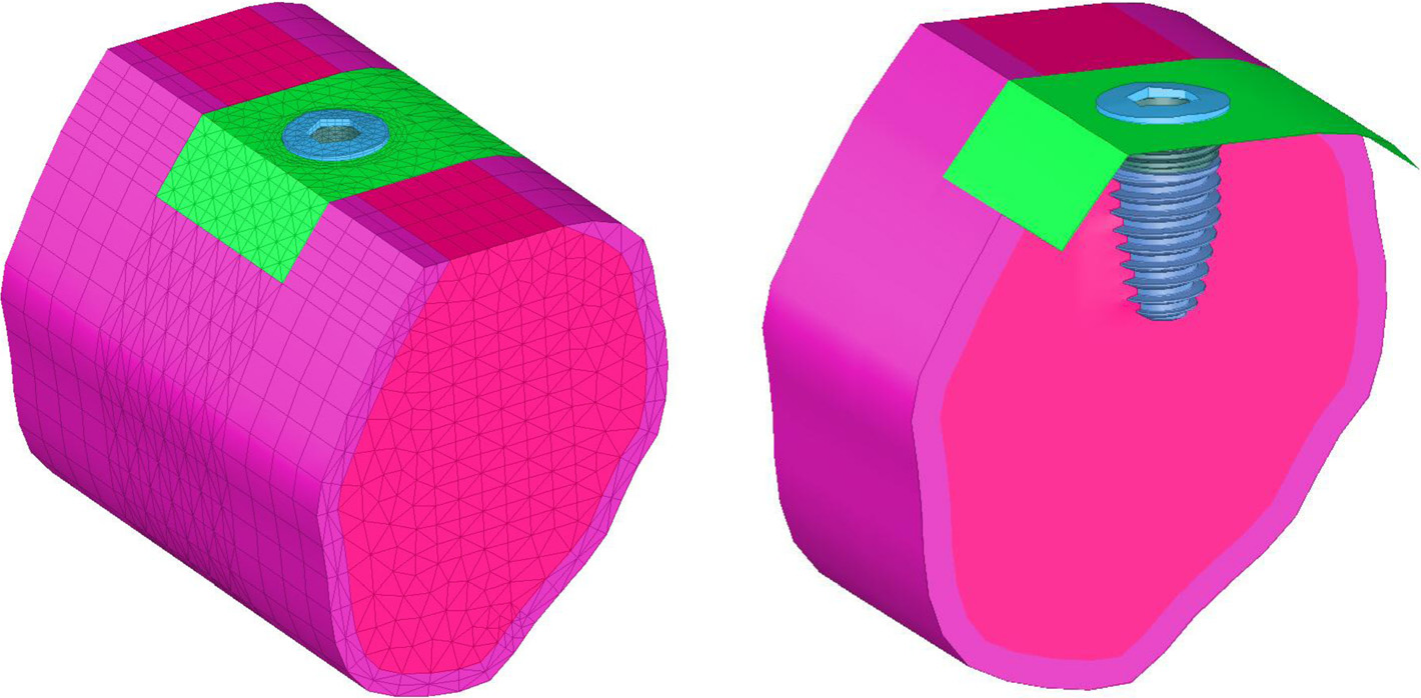
Class IV modifications

The saddle structure was constructed to wrap around the crest of the alveolar bone. In the mesiodistal direction, the length was fixed at 10 mm, and the dimension of the buccolingual length was changed from 2.5 to 5.5 mm by a 1-mm gradient from the center of the implant. The basic design for the analysis of FEA models is shown in Fig. 1. In class I modifications, the saddle was designed as 10 mm mesiodistally, 2.5 mm buccally, and 2.5 mm lingually from the center of the implant (Fig. 2). In class II modifications, the saddle was designed 10 mm mesiodistally, 3.5 mm buccally, and 3.5 mm lingually from the center of the implant (Fig. 3). The thickness of the saddle was 0.3 mm. In class III modifications, the saddle was designed as 10 mm mesiodistally, 4.5 mm buccally, and 4.5 mm lingually from the center of the implant (Fig. 4). In class IV modifications, the saddle was designed 10 mm mesiodistally, 5.5 mm buccally, and 5.5 mm lingually from the center of the implant (Fig. 5). With a 4.0-mm diameter and a 5.6-mm length, the implant was structured without modifications. The mechanical properties of the materials for the FEA model are listed in Table 1. Titanium was adopted for this finite elementary analysis.

**Table 1:** Mechanical properties of the materials used in the study

Material Young’s modulus Poisson ratio	
Cortical bone 14.8 0.30	
Cancellous bone 1.85 0.30	
Titanium 110 0.32	
Cobalt-chromium alloy 220 0.30	

### Applying the load

Because the loads to be applied with the mouse were not in the vertical direction of the implant, they had to be calculated in each direction. Therefore, we calculated them as below, with the force applied to the implant itself set as the vertical direction for force transfer and set it as L1. L2, corresponding to the direction of occlusion, was used for the oblique load on the implant. L3 was set for the horizontal directed force to the implant body.

- L2: oblique load: 300 N
- L1: vertical load: 300 * cos(10°) = 295.4 N
- L3: horizontal load: 300 * sin(10°) = 52.1 N

## Results

**Fig. 6:**
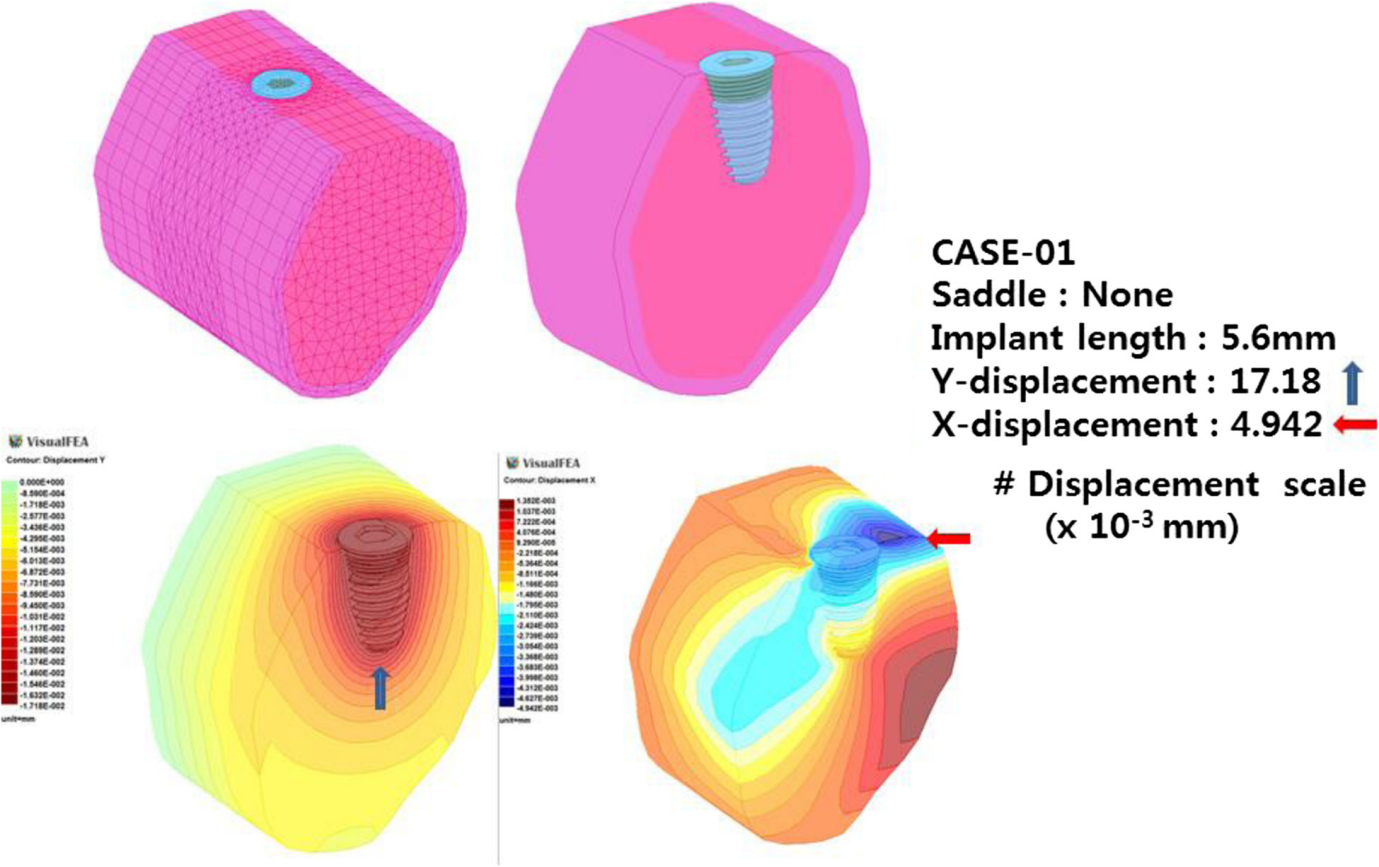
Conventional-type implant that is 4.0 mm in diameter and 5.6 mm in length without a saddle structure. Settlement of the surrounding bone and lateral displacement

**Fig. 7:**
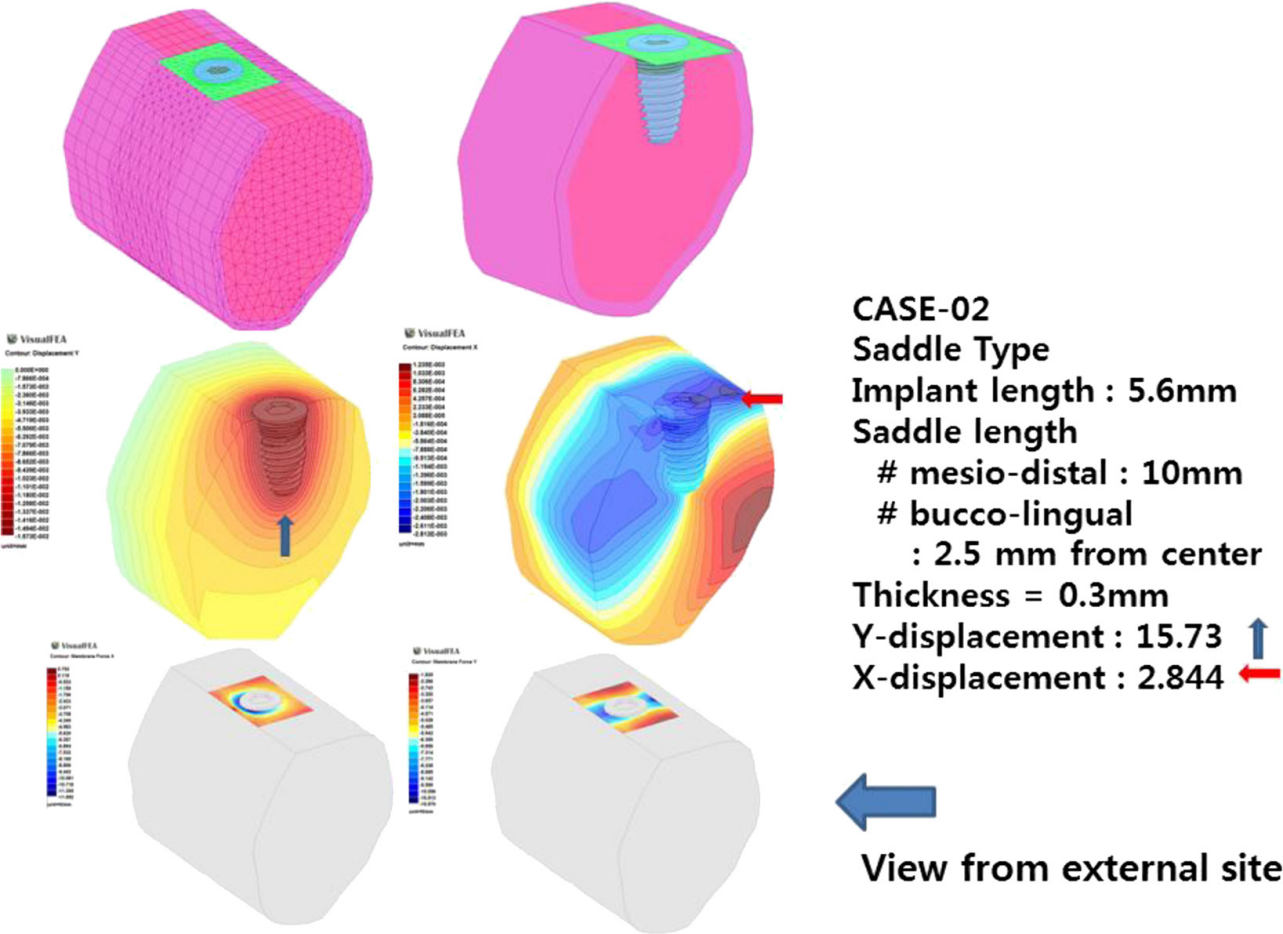
Results of a saddle-type implant with 2.5 mm wing and 5.6 mm length implant showing the lateral and vertical displacement of the surrounding bone

**Fig. 8:**
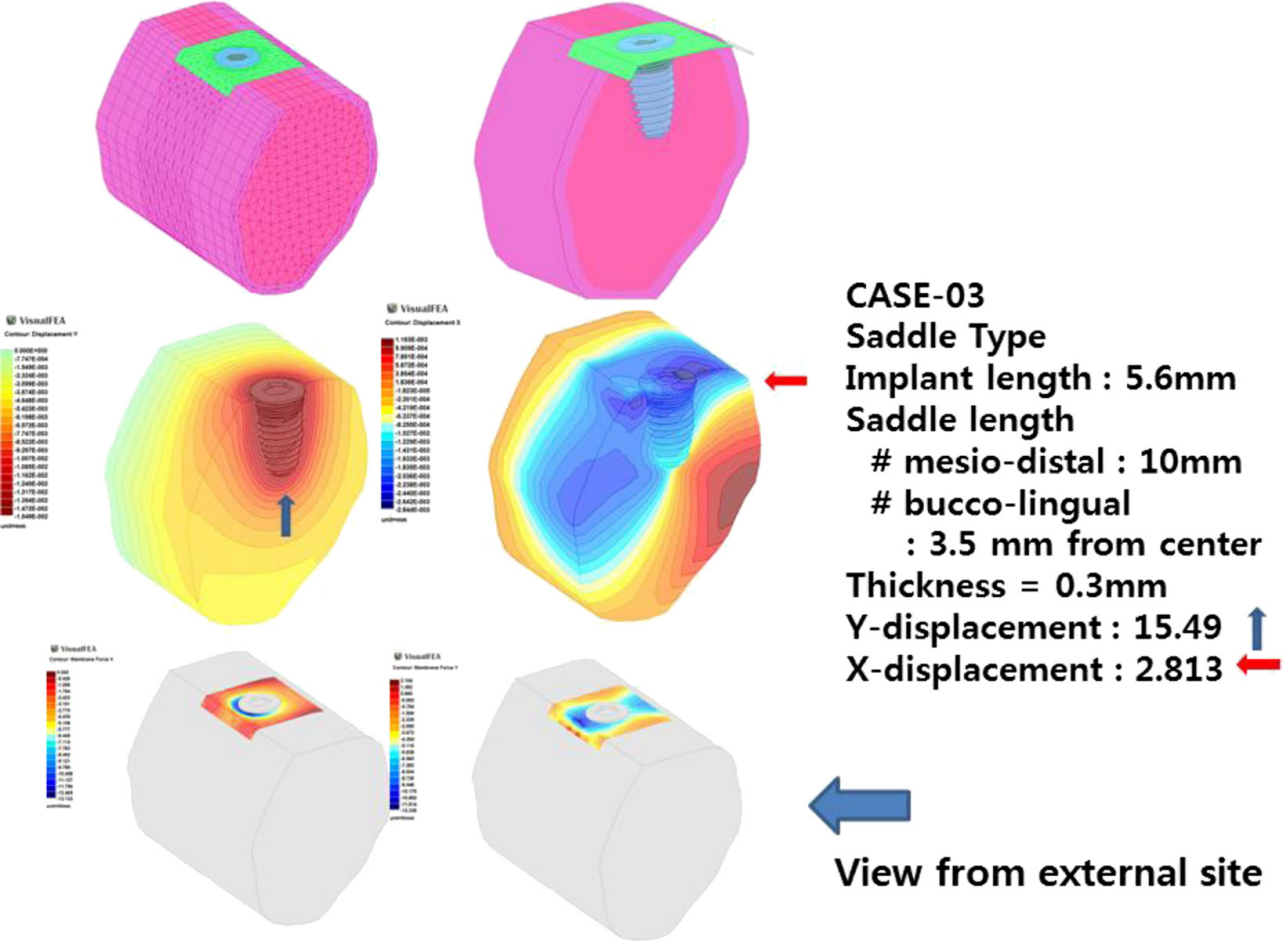
Results of a saddle-type implant with 3.5 mm wing and 5.6 mm length implant showing the lateral and vertical displacement of the surrounding bone

**Fig. 9:**
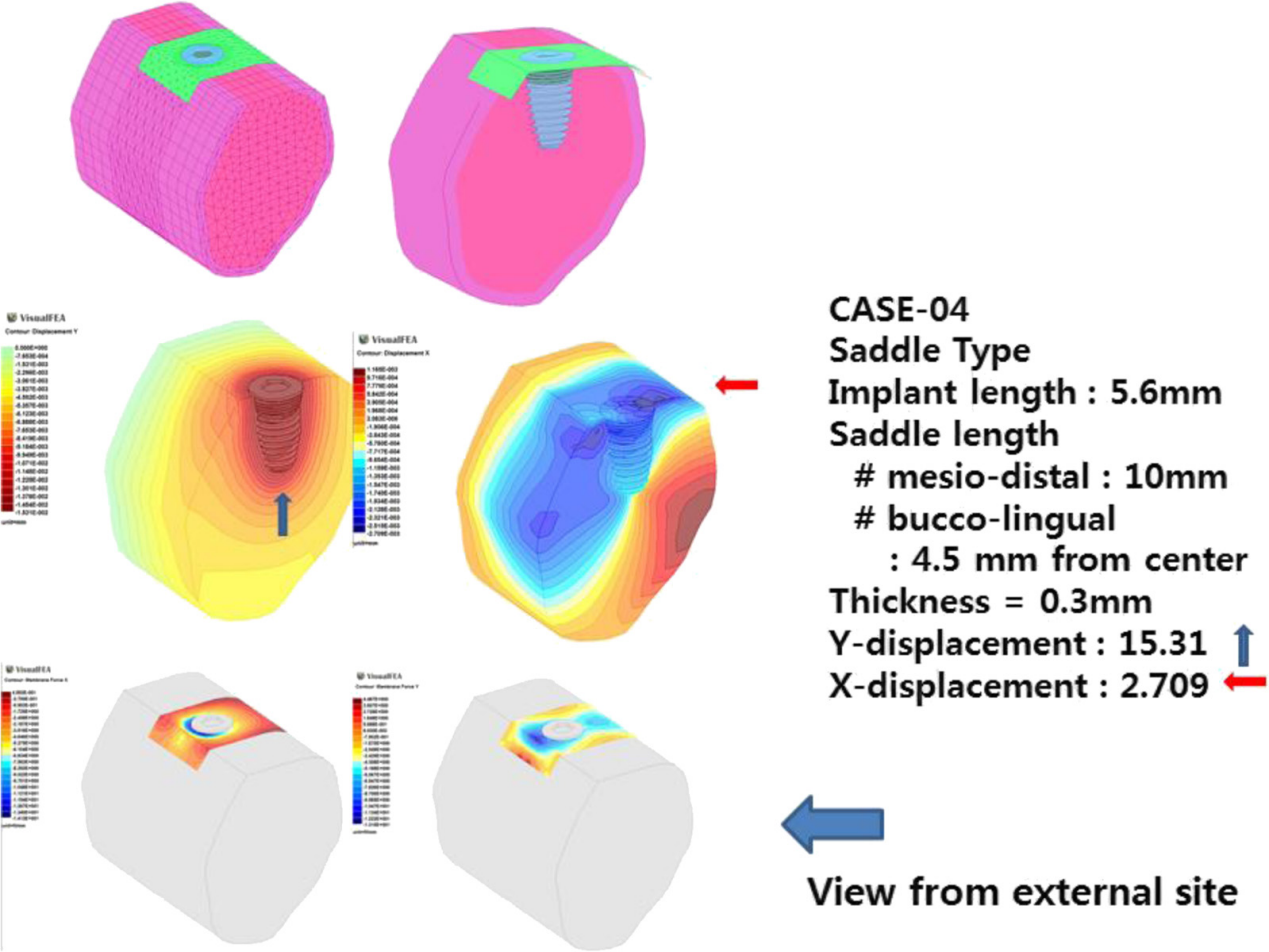
Results of a saddle-type implant with 4.5 mm wing and 5.6 mm length implant showing the lateral and vertical displacement of the surrounding bone

**Fig. 10:**
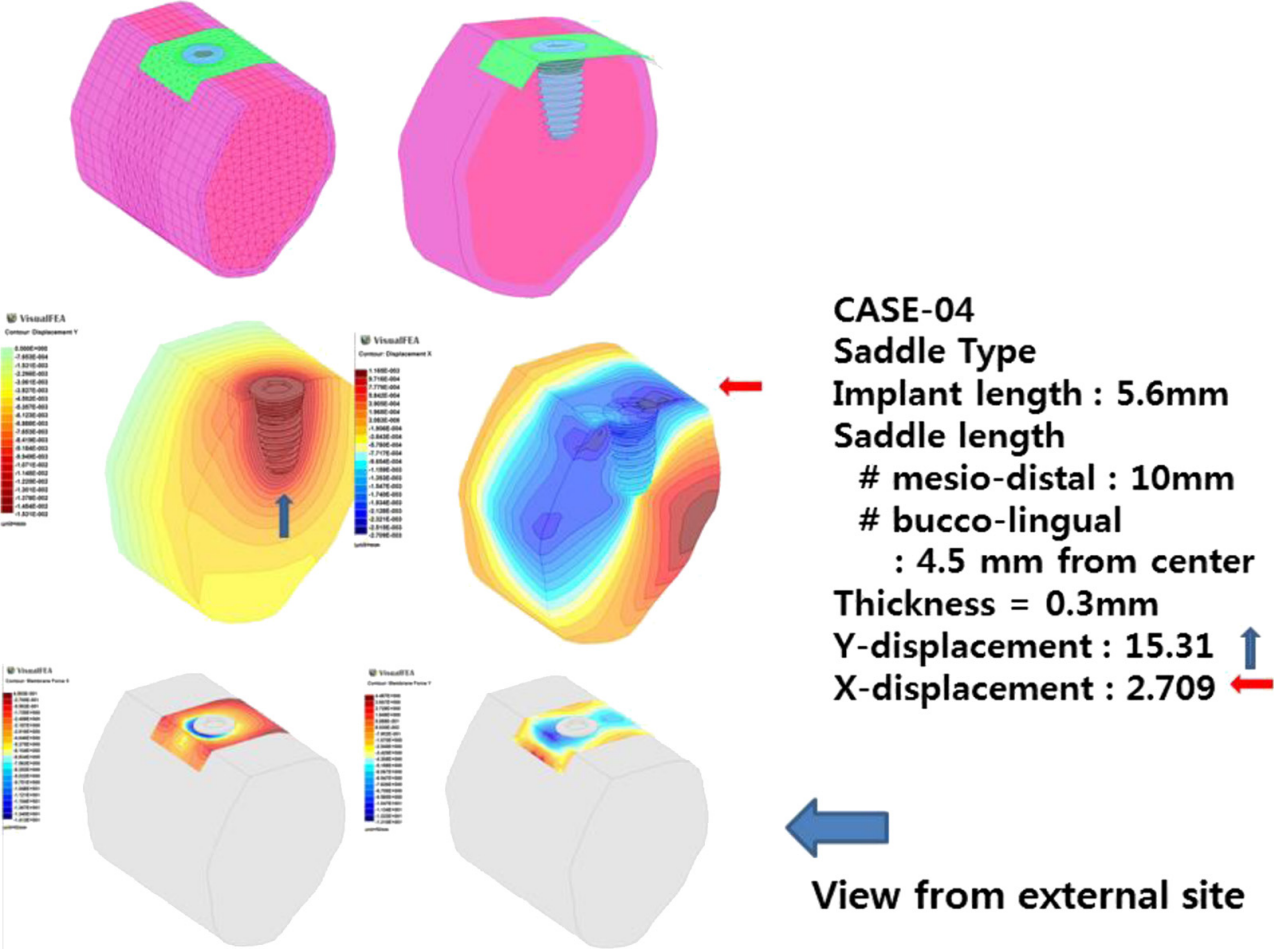
Results of a saddle-type implant with 5.5 mm wing and 5.6 mm length implant showing the lateral and vertical displacement of the surrounding bone

**Fig. 11:**
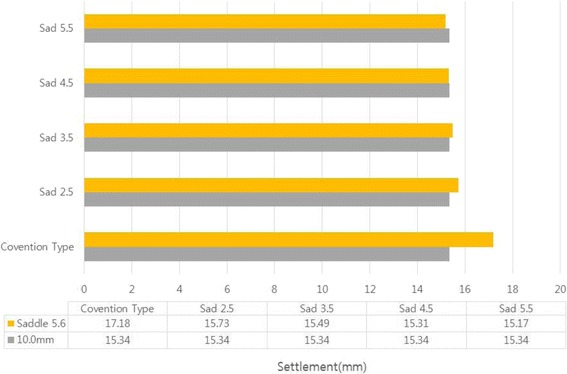
Results of comparing the settlement with a 10-mm length and 4.0-mm diameter conventional-type implant (*blue bar*) with a saddle-type implant (*orange bar*)

**Fig. 12:**
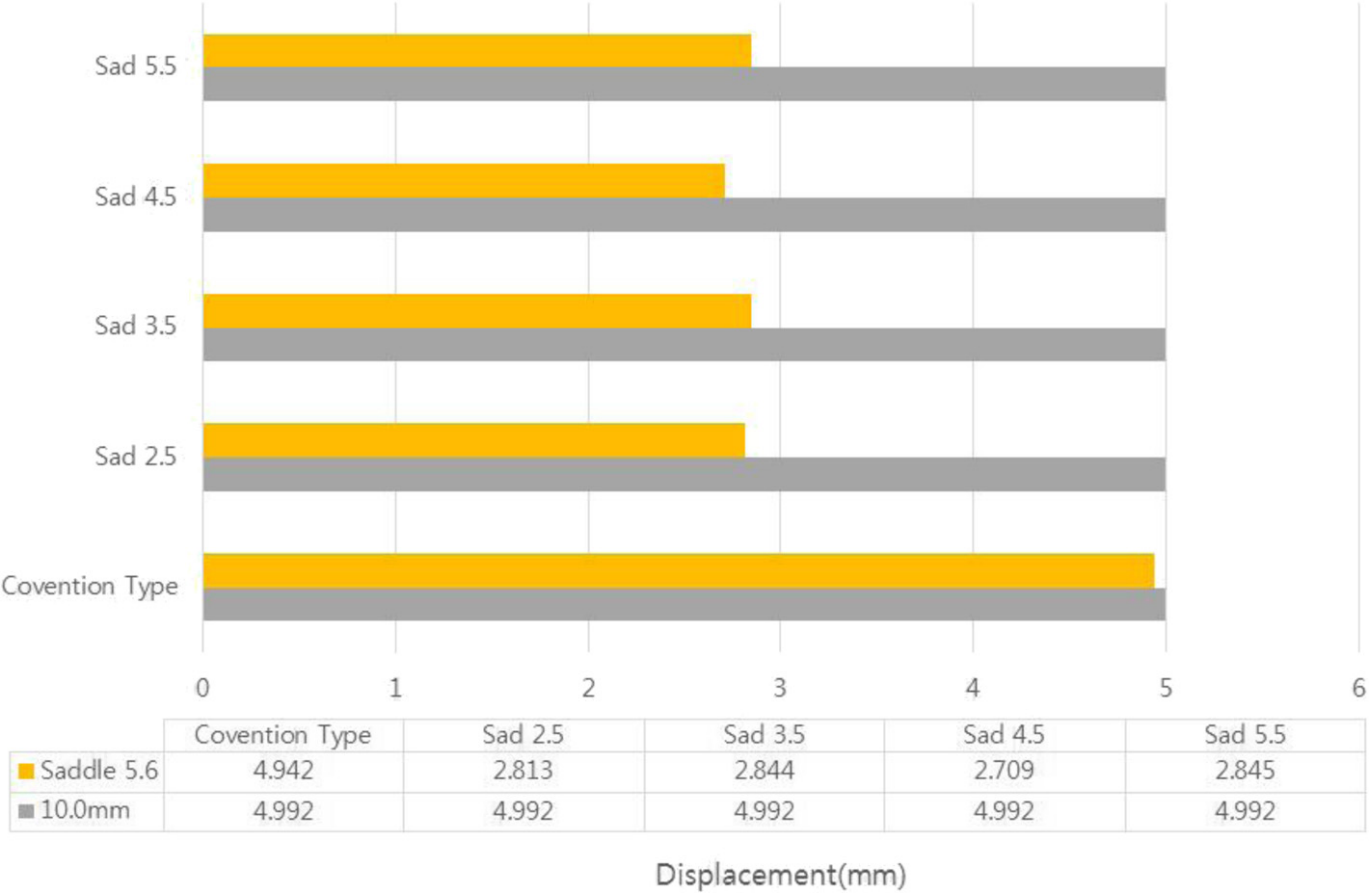
Results of comparing the lateral displacement with a 10-mm length and 4.0-mm diameter conventional-type implant (*blue bar*) with a saddle-type implant (*orange bar*)

The results of the FEA model studies are shown in Figs. 6, 7, 8, 9, 10, 11, and 12. The summary of the comparative data among the groups is shown in Table 2. The FEA results of CI (diameter, 4.0 mm; length, 5.6 mm) is shown in Fig 6. The settlement of the surrounding bone was 0.017 mm, and the lateral displacement was 0.005 mm. The FEA results of class I SI are shown in Fig. 7. The settlement was 0.016 mm, and the lateral displacement was 0.003 mm. The settlement of class II SI was 0.015 mm, and the lateral displacement was 0.003 mm (Fig. 8). The settlement of class III SI was 15.31 μm, and the lateral displacement was 2.71 μm (Fig. 9). The settlement of class IV SI was 15.17 μm, and the lateral displacement 2.56 μm (Fig. 10). Figure 11 demonstrates the results of comparing the settlement with CI (orange bar, length, 10 mm; diameter, 4.0 mm) to CI (blue bar). The settlement decreased by 4.5 mm from that with SI.

**Table 2:** Results of the FEA analysis

Item	Convention type		Saddle type (length 5.6 mm)			
	10 mm	5.6 mm	CASE1	CASE2	CASE3	CASE4
			2.5 mm	3.5 mm	4.5 mm	5.5 mm
Settlement (10^−3^ mm)	15.34	17.18	15.73	15.49	15.31	15.17
Displacement (10^−3^ mm)	4.992	4.942	2.813	2.844	2.709	2.556

The comparative results of the lateral displacement between SI (blue bar) and CI (orange bar) are demonstrated in Fig. 12. Lateral displacement with the SI was smaller than that with the CI.

## Discussion

During mastication, stress situations can occur due to occlusal force, and many authors have reported that alveolar bone resorption can occur due to overload. Therefore, it is very important to reduce the stress on the bone. In this study, the SI disseminated the stress more effectively than the CI. During implant treatment, surgeons encounter various situations restricting the implant. Especially when the alveolar bone is resorbed severely, only short implants can be used. However, because short implants are not very good at bearing occlusal force, wider implants are used. When wide and short implants are used, the remaining bone around the implant is reduced, and also, the crown implant ratio is increased, and longer leverage of superstructure can play a role in causing implant failure (fracture of implants, screw loosening or screw fracture, resorption of alveolar bone around the implant, etc.). To resolve such unfavorable conditions, the GBR technique is usually used for lateral and vertical bone augmentation. However, it requires the very skillful technique of an experienced surgeon, with a high risk of infection or wound dehiscence resulting in less effective bone augmentation.

The SI was introduced to the market in 1948 by Gershkoff [5], under the name sub-periosteal implant. However, it rapidly disappeared because of a high failure rate and early failures. This sub-periosteal implant was made by casting a Co-Cr-Va alloy, which is not as biocompatible as titanium and which requires a thick structure to bear the stress from occlusal force. Renouard and Nisad studied the difference in success rates by implant length and width, and they reported that short implants were usually used at posterior sites of the mandible and maxilla, with the implants positioned at these sites revealing lower survival rates [6].

A similar study was undertaken by Olate et al., but they reported that both the width and length affected the prognoses of implants. In this study, the shorter and narrower implants revealed higher failure rates [7]. Barikani also pointed out the limitations of using short and wide implants and recommended minimum bone removal, leaving as much bone remaining as possible [8]. Various types of studies have been performed on the designs of the necks of implants [9, 10].

One of the efforts undertaken to distribute force transmission was attempted to create a wing for the necks of implants. Wing-type implants showed much better results for stress distribution [11], and these implants are still developing today, with changes in the size, shape, pitch, etc. [12, 13]. The author designed an SI with a fixed length of with 5.6 mm, i.e., an ultra-short implant to solve the current problems when using CIs. The implant used with the SI was the same as the CI in shape, but its length was only 5.6 mm, and its diameter was 4.0 mm for the purpose of fixing the saddle structure to the underlying alveolar bone. This minimally invasive implant could reduce trauma on the bone, and its new design could be used in any compromised situation in which a CI cannot be inserted.

The stress distribution ability of newly designed implants was analyzed with an FEA model. Today, many studies have been performed on the structure of implants themselves and/or biomechanical analyses of the stress on implants, but there has been only limited study of the force distribution to the surrounding bone [14–17]. Stress on the implant due to occlusal force will be transmitted to the surrounding bone, and it will be affected by the implant shape, diameter, and the length. Qian et al. reported that, when lateral force was applied to implants, unfavorable stress was delivered to the surrounding bone, so it is important to use wider implants and to perform deeper insertion [18]. However, this type of implantation is not always possible because of anatomical limitations. Given that the structure of the implant is important to supporting stress [19], the new design might play an important role in obtaining successful outcomes for the rehabilitation of severely atrophied jaws.

## Conclusions

We compared the SI with the CI. The SI that was 5.6 mm in length demonstrated less lateral displacement than the CI that was 10 mm in length. In comparing the settlement, the SI showed more stability than the CI under occlusal stress. The limitation of the current study was that it consisted of the results of a simulation. Therefore, clinical trials should be performed.

## References

[1] Lin D, Li Q, Li W, Duckmanton N, Swain M (2010). Mandibular bone remodelling induced by dental implant. J Biomech.

[2] Lee JH, Frias V, Lee KW, Wright RF (2005). Effect of implant size and shape on implant success rates: a literature review. J Prosthet Dent.

[3] Lehman H, Casap N (2014). Rapid-prototype titanium bone forms for vertical alveolar augmentation using bone morphogenetic protein-2: design and treatment planning objectives. Int J Oral Maxillofac Implants.

[4] Jensen OT, Lehman H, Ringeman JL, Casap N (2014). Fabrication of printed titanium shells for containment of BMP-2 composite graft materials for alveolar bone reconstruction. Int J Oral Maxillofac Implants.

[5] Renouard F, Nisand D (2006). Impact of implant length and diameter on survival rates. Clin Oral Imp Res.

[6] Olate S, Lyrio MCN, Moraes M, Mazzonetto R, Moreira RWF (2010). Influence of diameter and length of implant on early dental implant failure. J Oral Maxillofac Surg.

[7] Barikani H, Rashtak S, Akbari S, Fard MK, Rokn A (2014). The effect of shape, length and diameter of implants on primary stability based on reasonance frequency analysis. Dent Res J.

[8] Velde T, Collaert B, Sennerby L, Bruyn H (2010). Effect of implant design on preservation of marginal bone in the mandible. Clin Implant Dentist Related Res.

[9] Chung JM, Jo KH, Lee CH, Yu WJ, Lee KB (2009). Finite element analysis of peri-implant bone stress influenced by cervical module configuration of endosseous implant. J Kor Acad Prosthodont.

[10] Park JW, Kim SG, Choi DW, Choi MR, Yoon YJ, Park JW (2012). Study of a “wing-type” implant on stress distribution and bone resorption at the alveolar crest. J Korea Acad Oral Maxillofac Surg.

[11] Tae Sun Lee (2014) Effects of the structural change of the implant on the stress distribution to the surrounding tissues and the stability of the implant. Thesis of Hallym University. June:1–28

[12] Gaviria L, Salcido JP, Guda T, Ong JL (2014). Current trends in dental implants. J Korean Assoc Oral Maxillofac Surg.

[13] Albrektsson T, Wennerberg A (2005). The impact of oral implants-past and future, 1966-2042. J Can Dent Assoc.

[14] Jimbo R, Halldin A, Janda M, Wennerberg A, Vandeweghe S (2013). Vertical fracture and marginal bone loss of internal-connection implants: a finite element analysis. Int J Oral Maxillofac Implants.

[15] Assunçᾶo WG, Gomes ÉA, Barᾶo VAR, Sousa EAC (2009). Stress analysis in simulation models with or without implant threads representation. Int J Oral Maxillofac Implants.

[16] Marcé-Nogué JM, Walter A, Gil L, Puigdollers AC (2013). Finite element comparison of 10 orthodontic microscrews with different cortical bone parameters. Int J Oral Maxillofac Implants.

[17] Simsek B, Erkmen E, Yilmaz D, Eser A (2006). Effects of different inter-implant distances on the stress distribution around endosseous implants in posterior mandible: a 3D finite element analysis. Medical Eng Physics.

[18] Qian L, Todo M, Matsushita Y, Koyano K (2009). Effects of implant diameter, insertion depth, and loading angle on stress/strain fields in implant/jawbone system: finite element analysis. Int J Oral Maxillofac Implants.

[19] Chang PK, Chen YC, Huang CC, Lu WH, Chen YC, Tsai HH (2012). Distribution of micromotion in implants and alveolar bone with different thread profiles in immediate loading: a finite element study. Int J Oral Maxillofac Implants.

